# Gentamicin Blocks the ACh-Induced BK Current in Guinea Pig Type II Vestibular Hair Cells by Competing with Ca^2+^ at the l-Type Calcium Channel

**DOI:** 10.3390/ijms15046757

**Published:** 2014-04-22

**Authors:** Hong Yu, Chang-Kai Guo, Yi Wang, Tao Zhou, Wei-Jia Kong

**Affiliations:** 1 Department of Otorhinolaryngology, Union Hospital of Tongji Medical College, Hua-Zhong University of Science and Technology, 1277 Jiefang Avenue, Wuhan 430022, China; E-Mails: yuhong_0706@163.com (H.Y.); ckguo2255@sina.com (C.-K.G.); entwy821@163.com (Y.W.); entzt2013@sina.com (T.Z.); 2 Institute of Otorhinolaryngology, Union Hospital, Tongji Medical College, Hua-Zhong University of Science and Technology, 1277 Jiefang Avenue, Wuhan 430022, China; 3 Key Laboratory of Neurological Disease, Ministry of Education, Tongji Medical College, Hua-Zhong University of Science and Technology, Wuhan 430022, China

**Keywords:** gentamicin, vestibular hair cells, big conductance calcium-dependent potassium channel, acetylcholine, calcium channel

## Abstract

Type II vestibular hair cells (VHCs II) contain big-conductance Ca^2+^-dependent K^+^ channels (BK) and l-type calcium channels. Our previous studies in guinea pig VHCs II indicated that acetylcholine (ACh) evoked the BK current by triggering the influx of Ca^2+^ ions through l-type Ca^2+^ channels, which was mediated by M2 muscarinic ACh receptor (mAChRs). Aminoglycoside antibiotics, such as gentamicin (GM), are known to have vestibulotoxicity, including damaging effects on the efferent nerve endings on VHCs II. This study used the whole-cell patch clamp technique to determine whether GM affects the vestibular efferent system at postsynaptic M2-mAChRs or the membrane ion channels. We found that GM could block the ACh-induced BK current and that inhibition was reversible, voltage-independent, and dose-dependent with an IC_50_ value of 36.3 ± 7.8 μM. Increasing the ACh concentration had little influence on GM blocking effect, but increasing the extracellular Ca^2+^ concentration ([Ca^2+^]_o_) could antagonize it. Moreover, 50 μM GM potently blocked Ca^2+^ currents activated by (−)-Bay-K8644, but did not block BK currents induced by NS1619. These observations indicate that GM most likely blocks the M2 mAChR-mediated response by competing with Ca^2+^ at the l-type calcium channel. These results provide insights into the vestibulotoxicity of aminoglycoside antibiotics on mammalian VHCs II.

## 1. Introduction

Aminoglycoside antibiotics are commonly used in developing countries due to their powerful broad-spectrum bactericidal ability, inexpensive cost and low allergenicity. However, widespread use of aminoglycosides has been restricted because of the incidence of serious side effects, such as nephrotoxicity, ototoxicity and muscle paralysis. Moreover, intratympanic application of gentamicin (GM), an ototoxic aminoglycoside, could be efficacious for treating vertigo in Meniere’s disease because it is more toxic to vestibular hair cells (VHCs) than cochlea hair cells [1–3]. Most studies have focused on the molecular mechanism of the hair cell damage by aminoglycosides. However, less electrophysiological evidence exists regarding the mechanism ofotoxicity, especially the vestibulotoxicity of aminoglycosides.

Many studies have demonstrated that aminoglycosides, including GM, can block many ion channels, such as voltage-gated calcium channels [4–7], mechanosensitive ion channels [8–12], and nicotinic ACh receptors (*n*AChRs) [13–15]. GM also has been shown to block the suppression effects of the medial olivocochlear efferent system in guinea pig [16–19]. It was previously reported that acute GM application can block the Ca^2+^ channel and the Ca^2+^-dependent K^+^ channel in semicircular canal hair cells of the frog [20]. However, in mammals, it remains poorly understood whether GM could affect the vestibular efferent system and whether GM could block ion channels present on VHCs.

ACh is the major inhibitory neurotransmitter of the vestibular efferent system [21]. Many studies have shown that mammalian VHCs express muscarinic ACh receptor (mAChR) subtypes [11,22,23] and nAChR subunits [24–26]. Our previous studies found that ACh could activate big-conductance Ca^2+^-dependent K^+^ channels (BK) mediated by M2 mAChRs and l-type calcium channels in guinea pig type II VHCs (VHCs II) [27–29]. Blanchet *et al*. [14] reported that GM could block the influx of Ca^2+^ through nAChRs in guinea pig outer hair cells. Therefore, we speculated that GM might have an effect on M2 AChRs in VHCs II and might block ion channels such as l-type Ca^2+^ channels and BK channels in VHCs II. The aim of this study was to determine whether GM could inhibit the vestibular efferent system at postsynaptic M2-mAChRs or the membrane ion channels such as BK channels and the l-type calcium channel in guinea pig VHCs II.

## 2. Results

### 2.1. GM Reversibly Blocked the ACh-Induced BK Current in Guinea Pig VHCs II in a Dose-Dependent and Voltage-Independent Manner

**Figure 1.**
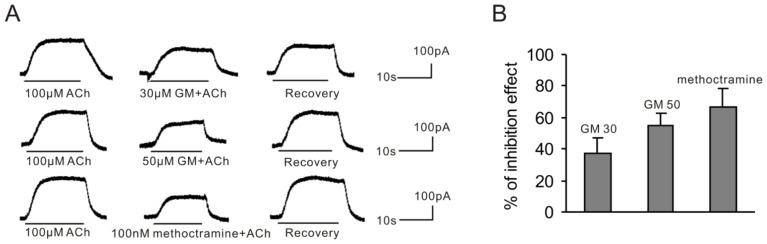
Effect of GM on the ACh-induced BK current. (**A**) Both 30 and 50 μM GM blocked the BK current evoked by 100 μM ACh. Methoctramine (100 nM) was used as a control. The above results were obtained from the same cell at −50 mV; (**B**) Bar histogram shows the percentage of blocking effect of 30 μM GM, 50 μM GM and 100 nM methoctramine on the current evoked by 100 μM ACh. Each point is the mean ± SD of 5–6 cells.

The effect of GM was assessed by comparing responses of VHCs II to applications of ACh with or without GM. Both 30 and 50 μM GM reversibly blocked ACh-induced BK currents in guinea pig VHCs II. Methoctramine (100 nM), an M2 selective AChR antagonist, was chosen as control (Figure 1A). The cell was washed with normal external solution after every drug application until it returned to normal. As shown in Figure 1B, 30 and 50 μM GM blocked ACh (100 μM)-induced BK currents by 37.1% ± 10.1% (*n* = 6) and 55.0% ± 7.4% (*n* = 6), respectively, while 100 nm methoctramine blocked it by 66.3% ± 12.4% (*n* = 5).

**Figure 2.**
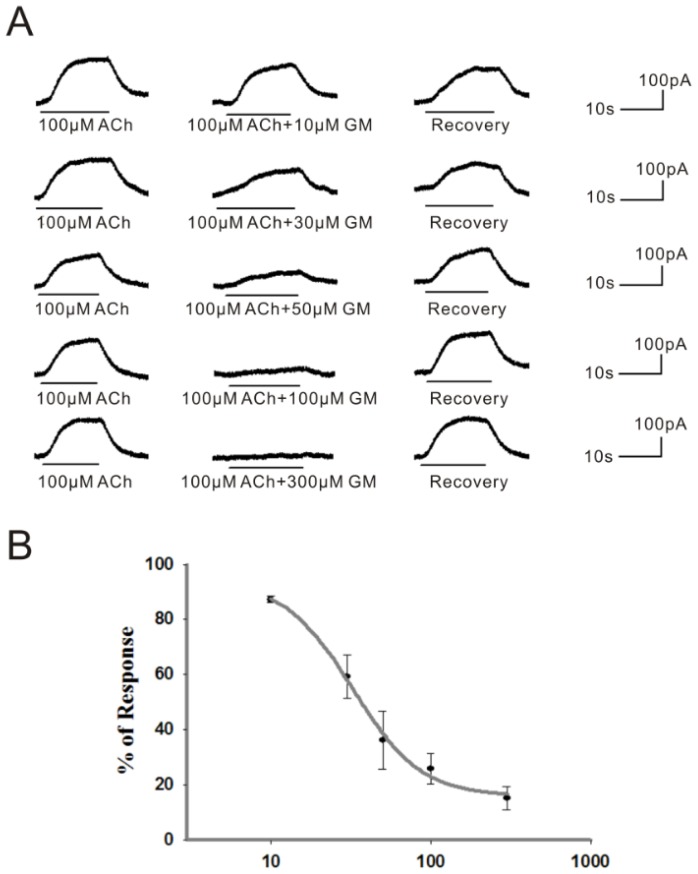
Different inhibition effects by various GM doses, and the dose-inhibition curve of GM. (**A**) With GM concentration increasing from 10 to 300 μM, the blocking effect increased gradually. The BK current was nearly completely blocked in the presence of 300 μM GM. The above results were obtained from the same cell at −50 mV; (**B**) The curve was derived by co-application of 100 μM ACh and increasing concentrations of GM. Only peak current values are plotted, expressed as the percentage of the peak control current evoked by ACh alone. Each point is the mean ± SD of 5 cells.

Next, the relationship between the inhibitory effect and the concentration of GM was studied. The dose dependency of the GM blocking effect was estimated by applying five different concentrations of GM, ranging from 10 to 300 μM, to the same VHC II (Figure 2A). The dose-inhibition curve of GM indicated that the dose for half-blocking response (IC_50_) was 36.3 ± 7.8 μM with a Hill coefficient near to one (Figure 2B).

**Figure 3.**
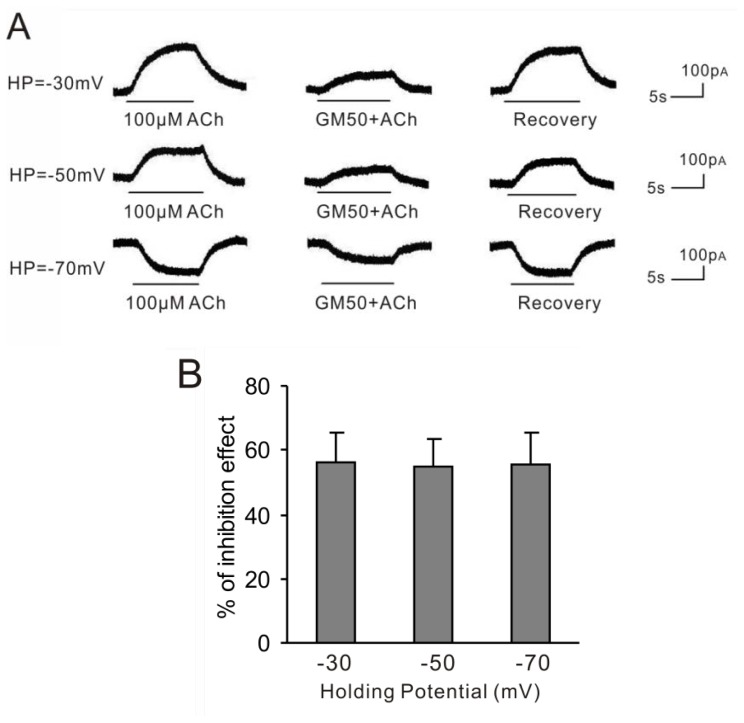
The blocking effects of GM at different holding potential. (**A**) Currents were sequential current traces evoked by 100 μM ACh (ACh100) alone or with 50 μM GM at holding potentials of −30, −50 and −70 mV. Results were obtained from the same cell; (**B**) Bar histogram showing the percentage of the blocking effect of 50 μM GM on 100 μM ACh-induced BK currents at three different holding potentials (−30, −50 and −70 mV). Each point is the mean ± SD of 5 cells.

We further studied the I/V relationship of BK currents induced by ACh supplemented with gentamicin. As shown in Figure 3, 50 μM GM blocked 100 μM ACh-induced BK current by 56.2% ± 9.1% (*n* = 5), 54.8% ± 8.9% (*n* = 5) and 55.8% ± 9.8% (*n* = 5) at holding potentials of −30, −50 and −70 mV, respectively. The homogeneity test of variance showed that there was no significant difference among three groups (*p* = 0.54). Using the one-way ANOVA, we found that the *F* value was 0.76 and the *p* value was 0.62, which indicated that there was no significant difference among the three groups. Therefore, GM inhibited ACh-induced BK currents in a voltage-independent manner.

### 2.2. Inhibition of GM Is not Affected by ACh Concentration

**Figure 4.**
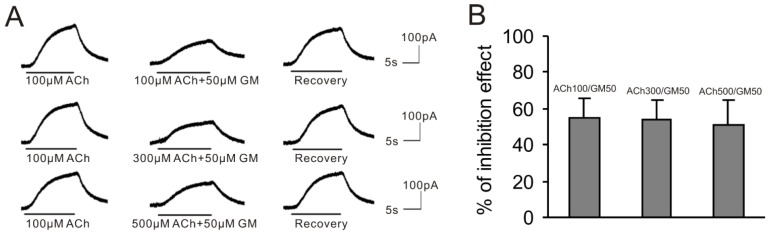
Increasing ACh concentration has little influence on GM inhibition effect. (**A**) The above currents were sequential current traces evoked by 100 μM ACh (ACh100) and different ACh concentrations (100, 300 and 500 μM) supplemented with 50 μM GM. The cell was clamped at −50 mV; (**B**) Bar histogram shows the percentage of the blocking effect of 50 μM GM supplemented with 100, 300 and 500 μM ACh on the current evoked by 100 μM ACh. Each point is the mean ± SD of 5 cells.

To determine whether GM could compete with ACh at its binding sites on the M2 mAChR, we increased the concentration of ACh with a fixed GM concentration. Our previous study demonstrated that the BK current nearly peaked at a concentration of 500 μM of ACh [28], indicating that M2 mAChRs of VHCs II were nearly saturated at that concentration. Therefore, in this study we tested three different solutions containing 100, 300 and 500 μM ACh with 50 μM GM. As shown in Figure 4, in the presence of these three ACh concentrations, 50 μM GM blocked the BK current by 55.0% ± 10.7% (*n* = 5), 54.0% ± 10.9% (*n* = 5) and 50.7% ± 13.7% (*n* = 5), respectively. The homogeneity test of variance showed that there was no significant difference among the three groups (*p* = 0.34). Using the one-way ANOVA, we found that the *F* value was 0.82 and the *P* value was 0.48, which indicated that there was no significant difference among the three groups. Therefore, increasing the ACh concentration did not affect the blocking effect of GM, and the GM inhibition was not due to competition with ACh at the M2 mAChR sites.

Our previous findings showed that the BK current evoked by 3 mM ACh was approximately 1.5 times of that activated by 100 μM ACh in guinea pig VHCs II [28]. Therefore, the M2 mAChRs of VHCs II may not be saturated at 100 μM ACh. Under this condition, increasing the ACh concentration above 100 μM would reduce the GM blocking effect by activating more M2 mAChRs and triggering more Ca^2+^ influx to activate more BK channels. However, our results showing that activation of more M2 mAChRs failed to increase K^+^ efflux were not consistent with this hypothesis. Therefore, another mechanism must be responsible for this inhibition.

### 2.3. Increasing the Extracellular Ca^2+^ Concentration Antagonizes GM Inhibition and GM Can Block Ca^2+^ Evoked by (−)-Bay-K8644

Since GM competed with Ca^2+^, we wondered whether the GM-mediated inhibitory effect on ACh-induced BK currents was mainly due to impairment of Ca^2+^ influx from the l-type Ca^2+^ channels in guinea pig VHCs II. Therefore, we increased the extracellular calcium concentration ([Ca^2+^]_o_) and then observed the blocking effect.

**Figure 5.**
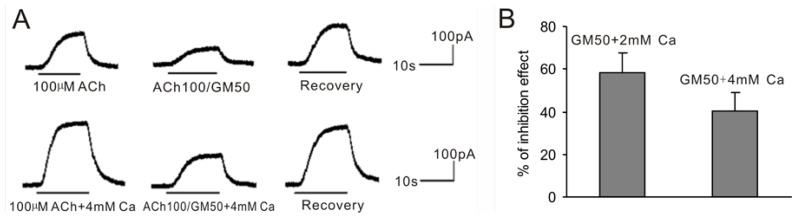
Increasing [Ca^2+^]_o_ antagonizes the GM inhibition effect of the BK current. (**A**) The sequential current traces evoked by 100 μM ACh alone or with 50 μM GM (ACh100/GM50) in the standard ([Ca^2+^]_o_ = 2 mM) or 4 mM [Ca^2+^]_o_ extracellular solution. The above currents were obtained from the same VHC II at −50 mV; (**B**) Bar histogram shows the percentage of inhibition effect of 50 μM GM on the BK current evoked by 100 μM ACh in standard extracellular solution or 4 mM [Ca^2+^]_o_ solution. Each point is the mean ± SD of 6 cells.

Our previous study showed that the ACh-induced BK current amplitude increased with the change of [Ca^2+^]_o_ from 2 to 4 mM, and that the current amplitude did not increase at concentrations higher than 4 mM [28]. Therefore, we decided to analyze the blocking effect of GM in 2 and 4 mM [Ca^2+^]_o_ solutions. As shown in Figure 5A,B, the blocking effect of 50 μM GM changed from 58.1% ± 9.7% (*n* = 6) to 40.3% ± 8.4% (*n* = 6, *p* < 0.05) upon increasing [Ca^2+^]_o_ from 2 to 4 mM. These results showed that GM could block BK currents in both normal and higher [Ca^2+^]_o_ solutions and the blocking effect was weaker in the elevated [Ca^2+^]_o_ condition. These results indicate that Ca^2+^ may compete with GM to antagonize GM inhibitory effect.

**Figure 6.**
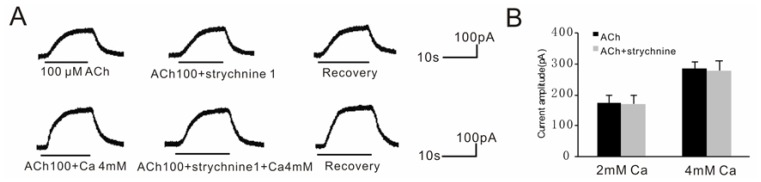
Effect of strychnine on ACh response in different [Ca^2+^]_o_ solutions. (**A**) BK currents evoked by 100 μM ACh were insensitive to 1 μM strychinine in both standard extracellular solution ([Ca^2+^]_o_ = 2 mM) and 4 mM [Ca^2+^]_o_ solution. These currents were obtained from the same VHC II at −50 mV; (**B**) A bar histogram shows the percentage of blocking effect of 1 μM strychnine on the BK current evoked by 100 μM ACh in standard extracellular solution or 4 mM solution. Each point is the mean ± SD of 5 cells.

It is known that nAChRs have high permeability to Ca^2+^ and can activate small-conductance Ca^2+^-dependent K^+^ channels in guinea pig VHCs II. Although our previous findings showed that the nAChRs were not involved in the BK currents recorded [27], it was still possible that Ca^2+^ influx though nAChRs was greater with higher [Ca^2+^]_o_. As shown in Figure 6, 1 μM strychnine, which is a potent nAChR antagonist, did not affect the BK current under normal [Ca^2+^]_o_. (*n* = 5, *p* = 0.46) or 4 mM [Ca^2+^]_o_. (*n* = 5, *p* = 0.37) solutions. These data indicated that there was no Ca^2+^ influx through nAChRs even in high [Ca^2+^]_o_ solution. The nAChR, which is not involved in BK currents, only affected the antagonism of elevated calcium concentrations by increasing Ca^2+^ influx. Therefore, we could rule out the involvement of nAChRs in the antagonism of GM blocking effect under elevated [Ca^2+^]_o_ conditions.

**Figure 7.**
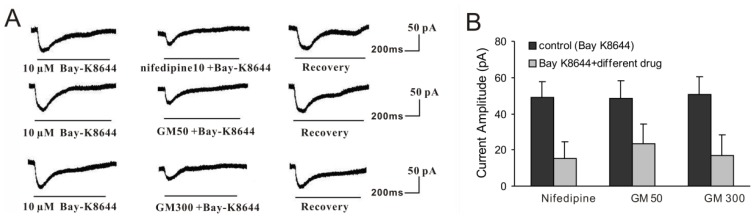
Effect of GM on (−)-Bay-K8644-activated current (HP = −50 mV). (**A**) The (−)-Bay-K8644-activated current was strongly blocked by 10 μM nifedipine. In addition, both 50 and 300 μM GM could block the current induced by 10 μM (−)-Bay-K8644; (**B**) Bar histogram showed the effects of 10 μM nifedipine, 50 μM GM, and 300 μM GM on the currents evoked by 10 μM (−)-Bay-K8644. Each point represents the mean ± SD of 5 cells.

The above data indicated that GM blocked BK currents by impairing Ca^2+^ influx, which was not mediated by *n*AChRs. Our previous studies have reported that ACh evoked BK currents by triggering Ca^2+^ influx through l-type Ca^2+^ channels [27]. Therefore, GM likely decreased Ca^2+^ influx through l-type Ca^2+^ channels. To study the direct effect of GM on l-type Ca^2+^ channels, we assessed whether GM affected the calcium current evoked by (−)-Bay-K8644 (the l-type Ca^2+^ channel agonist). We first verified the (−)-Bay-K8644-activated inward current by applying nifedipine (a Ca^2+^ channel blocker). As shown in Figure 7, the currents evoked by 10 μM (−)-Bay-K8644 in guinea pig VHCs II was potently reduced by 10 μM nifedipine to 25.1% ± 9.8% (*n* = 5) as expected. The results showed that the (−)-Bay-K8644-activated Ca^2+^ current was blocked by 50 μM GM (48.4 ± 10.1 *vs.* 23.2 ± 11.2 pA, *p* < 0.05, *n* = 5; Figure 7) and 300 μM GM (50.4 ± 9.8 *vs.* 17.0 ± 11.5 pA, *p* < 0.05, *n* = 5; Figure 7) compared to control, respectively. These results indicated that GM decreased the influx of Ca^2+^ through the l-type Ca^2+^ channel.

### 2.4. BK Current Evoked by NS1619 Insensitive to 50 μM GM and Only Slightly Blocked by 300 μM GM

**Figure 8.**
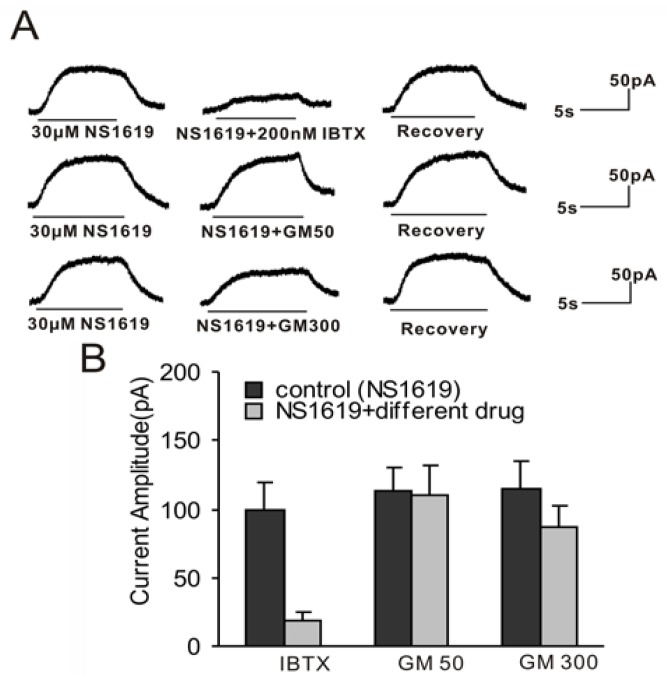
Effect of GM on NS1619-activated current (HP = −50 mV). (**A**) The NS1619-activated current was strongly blocked by 200 nM IBTX. In addition, 50 μM GM could not block the current induced by 30 μM NS1619, while 300 μM inhibited it slightly; (**B**) Bar histogram showed the effects of 200 nM IBTX, 50 μM GM, and 300 μM GM on the current evoked by 30 μM NS1619. Each point represents the mean ± SD of 5 cells.

In order to determine whether GM has a direct blocking effect on BK channels in guinea pig VHCs II, we observed the effect of GM on BK currents activated by NS1619 (a BK channel activator). We verified the NS1619-activated outward current by applying IBTX (a BK channel blocker). As expected, the BK current induced by 30 μM NS1619 was potently blocked by 200 nM IBTX to 19.1% ± 7.8% (*n* = 5) (Figure 8). Therefore, the current activated by NS1619 was the BK current. We also found that the NS1619-activated BK current was not sensitive to 50 μM GM (control 113 ± 16.7 pA, 50 μM GM + NS1619 109.5 ± 22.2 pA, *p* = 0.26, *n* = 5; Figure 8). Moreover, 300 μM GM could only slightly block the current (control 115.2 ± 19.8 pA, 300 μM GM + NS1619 87.4 ± 15 pA, *p* = 0.03, *n* = 5; Figure 8). These results indicate that GM may have a slight direct blocking effect on the BK channel at high concentrations.

## 3. Discussion

It has been reported that GM can damage efferent nerve endings on VHCs [30,31], but the physiological mechanism of this damage is still unclear. In the present study, using the whole-cell patch clamp technique, we demonstrated that GM could reversibly block the ACh-induced BK current in guinea pig VHCs II in a dose-dependent and voltage-independent manner, which indicated that acute GM application could inhibit the vestibular efferent system at the level of the postsynaptic membrane in mammalian VHCs.

**Figure 9.**
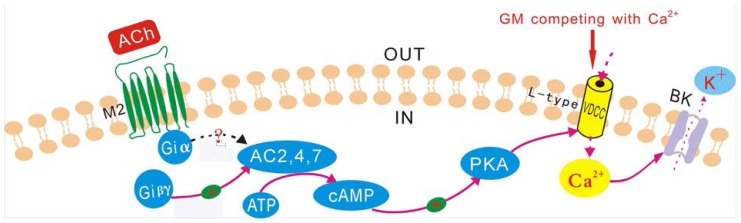
The l-type calcium channel was the probable blocking site of GM. The signal transduction pathway of the ACh-induced BK current in guinea pig VHCs II and the probable blocking site of GM on the pathway. GM probably blocks the ACh-induced BK current mainly by competing with Ca^2+^ at the l-type calcium channel. **+**: excitation.

Our previous studies demonstrated that the BK channel and the l-type calcium channel were co-located in guinea pig VHCs II [29]. ACh could evoke the BK current by triggering the Ca^2+^ influx from l-type calcium channels in guinea pig VHCs II mediated by M2 mAChRs [27]. Since GM could block the ACh-induced BK current in isolated VHCs II, it would affect at least one site of the signal transduction pathway. The acute application of GM in this study likely inhibited receptors or ion channels present on the plasma membrane of cells. As shown in Figure 9 [27], in the signal transduction pathway of the ACh-induced BK current, there were only three possible blocking sites on the membrane: the M2 mAChR, the l-type Ca^2+^ channel, and the BK channel.

First, if GM could compete with ACh at the M2 mAChR, increasing ACh concentration would reduce the GM inhibitory effect of BK currents. It was previously reported that M2 mAChRs of VHCs II were not saturated at 100 μM ACh [28], therefore, increasing the ACh concentration to 300 and 500 μM would activate more M2 mAChRs. However, the present study showed that increased M2 mAChR activation did not lead to reduction of GM inhibition, which eliminated the hypothesis of direct competition of GM with ACh at the M2 mAChR. Therefore, GM may block BK currents by affecting calcium influx or directly blocking BK channels.

The current findings demonstrated that increasing [Ca^2+^]_o_ could antagonize GM blocking effect, which indicated that GM may block the BK current by impairing calcium influx. It has been reported that GM could impair the calcium influx from the calcium channels [6,31,32] and the specific binding sites at the nAChRs [14]. In guinea pig VHCs II, nAChRs and calcium channels coexist, so increasing the extracellular Ca^2+^ concentration alleviated GM blocking effect through both or only one of them. Our previous findings showed the nAChRs were not involved in the activation of BK channels and BK currents evoked by ACh was insensitive to strychnine [27]. The present results showed that the ACh-induced BK currents were not affected by strychnine even in the higher [Ca^2+^]_o_ solution. Recently, we verified that nAChRs were deactivated in collagenase IA-isolated VHCs II [33]. Therefore, the effect of elevated [Ca^2+^]_o_ on the GM blocking effect was not due to nAChRs, but rather the l-type Ca^2+^ channel. To obtain more direct evidence of GM competing with Ca^2+^, we recorded the Ca^2+^ current evoked by (−)-Bay-K8644 and observed the direct effect of GM on it. The results showed that both 50 and 300 μM of GM potently inhibited the Ca^2+^ currents, indicating that GM could block l-type Ca^2+^ channels and decrease the influx of Ca^2+^. GM slightly blocked NS1619-activated BK currents at a high concentration of 300 μM, so the BK channel might be the blocking target of GM. Some studies have also shown that aminoglycosides could directly block the K^+^ channel [34–37]; however, the BK channel would not be the main blocking site. One reason was because the IC_50_ of GM to the ACh-induced BK current was 36.1 ± 7.8 μM, but 50 μM GM did not block the NS1619-activated BK current and only very slightly inhibited it at a concentration of 300 μM. The other important reason was that it could not explain the reduction of GM blocking effect with increasing [Ca^2+^]_o_. Therefore, the direct blocking of BK channels might be a very small explanation, but is not the major mechanism.

Our previous study demonstrated that the activation of ACh-induced BK current was mainly dependent on external Ca^2+^ influx through l-type calcium channels, but not the release of intracellular Ca^2+^ stores [28]. Based on the above results, the GM blocking effect was likely due to competing with Ca^2+^ at the l-type calcium channel, which impaired the calcium influx to diminish the BK current. This provided a good explanation for the absence of increased K^+^ efflux even after the activation of more M2 mAChRs. Martini *et al*. [20] have reported that GM also blocked the Ca^2+^-dependent K^+^ current by impairing the Ca^2+^ influx in semicircular canal hair cells of the frog.

Our results also showed that GM reversibly blocked the ACh-induced BK current in guinea pig VHCs II in the micromolar range. GM also blocked the ACh-induced small-conductance calcium-dependent potassium current in guinea pig outer hair cells [14]. However, the IC_50_ (36.3 μM) of GM to the ACh-induced BK current mediated by the M2 mAChR was higher than GM (5.5 μM) to ACh-evoked K^+^ currents in outer hair cells at the nAChR. This may be due to differences in cell type or cholinergic receptor type.

## 4. Methods

### 4.1. Ethics Statement

The Institutional Animal Care and Use Committee of Tong-ji Medical College approved this animal experiment on 1 June 2010 (IACUC Number: 289).

### 4.2. Animal Procedures and VHCs II Preparation

Collagenase type IA-dissociated vestibular hair cells were isolated as previously described [27–29]. First, young guinea pigs (weighing 250–300 g, 6–10 weeks-old) were deeply anesthetized by intramuscular injection of 0.3 mL of a mix of ⅓ xylazine (2%, Rompum, Bayer, Leverkusen, Germany) and ⅔ ketamine hydrochlorate (50 mg/mL, Ketalar, Parke-Davis, L’Arche, France), and decapitated. Then we removed vestibular epithelium (three semicircular canals and two otolithic organs) and incubated it for 5 min at room temperature (20–24 °C) with 0.2 mg/mL collagenase IA in a low Ca^2+^ and Mg^2+^-free balanced salt solution (137 mM NaCl, 5.4 mM KCl, 0.1 mM CaCl_2_, 0.2 mM Na_2_HPO_4_, 0.4 mM KH_2_PO_4_, 10 mM glucose (pH 7.2)). Next, the low calcium solution was replaced with normal external solution containing 2 mM CaCl_2_ to stop the enzymatic action. VHCs were isolated by mechanical dissociation and placed on the bottom of the experimental chamber, which was coated with rat collagen. We identified VHCs II by the cylindrical shape and absence of a distinct neck region [38].

### 4.3. Electrophysiology

The ACh-induced BK currents were recorded in the whole-cell configuration, using an Axon-200B patch clamp amplifier (Axon Instruments, Foster City, CA, USA). Patch electrodes were fabricated from thick-walked borosilicate glass capillaries, using a Model P-97 electrode puller (Sutter Instrument Company, Novato, CA, USA). Electroderesistances were maintained between 3 and 6 MΩ when filled with the internal solution, as described below. Records were low-pass filtered at 5 kHz with a four-pole Bessel filter. After gigaseal formation onto the basolateral membrane of VHCs and membrane disruption, the membrane capacitance was 4.8 ± 1.7 pF on average (*C*m, *n* = 6) and the series resistance (RS, 6–15 MΩ) was compensated by up to 80%. The drug was administered to a patched cell at a holding potential of −50 mV.

The components of the external solution were as follows: 150 mM NaCl, 5 mM KCl, 2 mM CaCl_2_, 1 mM MgCl_2_, 5 mM glucose, and 10 mM HEPES (pH 7.2). The components of the internal solution were as follows: 150 mM KCl, 2 mM MgCl_2_, 3 mM Na_2_ATP, 0.1 mM EDTA and 10 mM HEPES (pH 7.2). The KCl was replaced by CsCl when the calcium current was recorded.

### 4.4. Drug Application

All drugs were purchased from Sigma (St. Louis, MO, USA). ACh, gentamicin (GM), methoctramine (an M2 selective AChR antagonist), strychnine (a selective *n*AChR inhibitor), and iberiotoxin (IBTX, a selective BK channel blocker) were directly dissolved in the external solution. NS1619 (a BK channel activator) was formulated as a 10 mM stock solution in DMSO (Sigma, St. Louis, MO, USA). (−)-Bay-K8644 (the l-type calcium channel agonist) and nifedipine (a calcium channel blocker) were formulated as 10 mM stock solutions in DMSO and diluted for use. The test solutions were dissolved daily before use and applied to cells by a gravity-delivered linear barrel microperfusion system as previously described [27–29]. The microperfusion system was composed of a series of fused silica tubes (eight tubes; outer diameter, 500 μm; internal diameter, 200 μm) connected to a series of independent reservoirs. The tip of the tube was placed approximately 100 to 150 μm from the cells. This microperfusion system was manipulated by shifting the tubes horizontally with a Leitz micromanipulator (ACS01, Leitz Corp., Wetzlar, Germany).

### 4.5. Data Analysis

Data were analyzed and plotted by using the pCLAMP8.1 Clampfit 8.1 software (Axon Instruments, Foster City, CA, USA) and SigmaPlot 9.0 (Systat Software, Richmond, CA, USA). Results were presented as the mean ± SD. Statistical significance was determined using the Student’s *t* test to compare the means between two groups, and one-way analysis of variance (ANOVA) to compare the means among more than two groups. Differences were considered to be significant if *p* < 0.05; all the differences listed were statistically significant, unless stated otherwise.

## 5. Conclusions

In conclusion, our findings indicate that acute GM application could block the ACh-induced BK current in guinea pig VHCs II by competing with Ca^2+^ at the l-type calcium channel, which results in the decrease of Ca^2+^ influx and the subsequent reduction of the BK current. Thus, the effect of GM on *n*AChRs in guinea pig VHCs II should be further investigated.
